# A novel tool for assessing psychomotor proficiency in laparoscopic cholecystectomy using simulation-based training

**DOI:** 10.1007/s00464-025-11708-2

**Published:** 2025-04-18

**Authors:** Erin Kim, Marius J. Tchinde, Deborah M. Rooney, Blessing N. Ngam, R. Luke Rettig, Christopher L. Gross, Mark J. Snell, Melanie L. Barnard, Kevin El-Hayek, David R. Jeffcoach, Grace J. Kim

**Affiliations:** 1 University of Michigan Medical School, Ann Arbor, MI USA; 2 Mbingo Baptist Hospital, Mbingo, Cameroon; 3 Department of Learning Health Sciences, University of Michigan Medical School, Ann Arbor, MI USA; 4 Kaiser Permanente South Bay Medical Center, Harbor City, CA USA; 5 University of North Carolina at Chapel Hill, Chapel Hill, NC USA; 6 Southern Illinois University, Carbondale, IL USA; 7 The MetroHealth System, Case Western Reserve University School of Medicine, Cleveland, OH USA; 8 Department of Surgery, University of California San Francisco Fresno, Fresno, CA USA; 9 Department of Surgery, University of Michigan, 1500 E Medical Center Drive, SPC 5331, Ann Arbor, MI 48109 USA

**Keywords:** Laparoscopy, Simulation, Global surgery, Cholecystectomy, Psychomotor

## Abstract

**Introduction:**

Laparoscopy is underutilized in lower- and middle-income countries (LMICs) due to limited access to training opportunities. Laparoscopic cholecystectomy is the gold standard for treating gallbladder disease in high-income countries (HICs), yet the open approach predominates in LMICs. A low-cost, simulation-based educational module for teaching laparoscopic cholecystectomy was developed by an international collaboration. A novel tool for verifying proficiency (CHOLE-VOP), incorporating a procedural checklist, a global rating scale (GRS), and a final competency rating, was designed and piloted to evaluate psychomotor skill acquisition in laparoscopic cholecystectomy.

**Methods:**

Fifty-two users completed the learning module, submitted a video recording of their performance on the tool, and performed self- and peer-assessment of videos using the CHOLE-VOP. A Kruskal–Wallis test was used to assess the CHOLE-VOP’s ability to differentiate psychomotor performance across three experience levels [novice (no laparoscopic experience), intermediate (1–30 cases), and expert (> 30 cases)] and between settings (LMICs vs. HICs). Inter-rater agreement was measured between self-assessment and peer-assessment, across reviewer reviewer experience levels.

**Results:**

Among users [novices (14), intermediates (18), experts (17), unknown (3)], checklist scores significantly increased from novice (*M* = 28.16) to intermediate (*M* = 31.33) and expert (*M* = 32.66), *P* < 0.001. Both GRS and final ratings effectively discriminated between experience levels, *P* < 0.001. LMIC users had higher checklist scores (32.41 vs. 29.35, *P* = 0.008) and GRS scores (17.54 vs. 15.14, *P* = 0.002). Inter-rater agreement between self- and peer-assessments was moderate (ICC = 0.52), with poor agreement for novices (ICC = 0.24), who tended to overestimate their performance, and good agreement for intermediate users (ICC = 0.79).

**Conclusion:**

The CHOLE-VOP successfully discriminated between experience levels in a simulated laparoscopic cholecystectomy training module. LMIC users outperformed HIC users in select skill parameters. Self-assessments, particularly among novices, showed limited concordance with peer-assessments.

**Graphical abstract:**

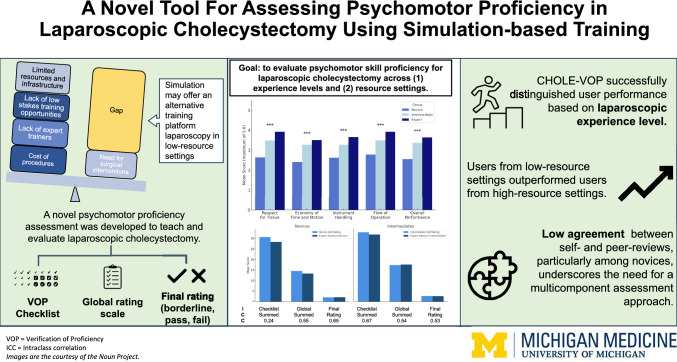

Access to quality surgical care in lower- and middle-income countries (LMICs) remains a critical challenge, with the gap magnified in minimally invasive surgery [1, 2]. Laparoscopic cholecystectomy consists of 21% of elective general surgical procedures at ACS-NSQIP–participating hospitals and is one of the most common operations among general surgeons [3]. Since the first laparoscopic cholecystectomy in 1987, over 90% of cholecystectomies are now being performed laparoscopically in the United States (US) [4, 5]. The incidence of gallbladder disease is rising in LMICs, due to the increasing prevalence of metabolic disorders like obesity and diabetes, driven in part by urbanization and poor dietary habits [6]. Despite the growing need for laparoscopic cholecystectomy in LMIC settings, a study of the operative logs of general surgery trainees from the College of Surgeons of East, Central and Southern Africa (COSECSA) region showed that only 23% of all cholecystectomies were laparoscopic, presenting a gap [1].

Laparoscopy offers numerous advantages over open surgery, including shorter hospital stays, fewer postoperative complications including surgical site infections and incisional hernias, and reduced patient mortality. The benefits of a minimally invasive approach are even magnified in LMICs, where early return to the workforce is crucial for economic vitality, due to the lack of infrastructure to support employee benefits, such as sick leave. Despite the well-known benefits of laparoscopy, widespread adoption of laparoscopy is limited by the lack of structured training opportunities and educational content to teach fundamental laparoscopic skills in an accessible and feasible approach [7]. Thus, there is an urgent need for alternative training methods that can equip LMIC surgeons with the necessary skills to perform laparoscopic cholecystectomy safely.

Simulation-based training holds the potential to bridge educational gaps in LMICs. Self-directed, online programming provides opportunities for asynchronous training with embedded assessments, which circumvents the need for a physical instructor at this stage [7–9]. Additionally, simulation allows for repetitive, safe practice in a low-stake environment, without the possibility of patient harm. Prior studies have shown that learners who trained on simulation-based training platforms made fewer intraoperative errors and had lower operative times, demonstrating increased accuracy and efficiency in the operating room (OR) [10–12]. Given the widening gap in surgical education between LMICs and high-income countries (HICs), a training platform that supports skill development across all experience levels holds significant value.

ALL-SAFE is an open-source, online laparoscopic surgery training platform designed for varied-resource settings. Developed through a collaboration between surgeons from Africa and the US, in partnership with the Pan-African Academy of Christian Surgeons (PAACS), the platform integrates both cognitive and psychomotor skill development. It features (1) a case-based learning exercise and (2) a low-cost, self-assembled box trainer, paired with an expert video tutorial for psychomotor skills. Users practice simulated procedures within the simulator and upload a video of their best performance on the platform for self, peer and AI review [13–16]. To date, ALL-SAFE has successfully released five procedural modules covering the laparoscopic management of ectopic pregnancy, appendicitis, penetrating thoracoabdominal injury, and Meckel’s diverticulum. Previous studies have shown that users of all experience levels demonstrated increased confidence, competence, and psychomotor skill acquisition following training in salpingostomy and appendectomy [13–16]. This study aims to assess psychomotor skill improvement across users from different experience levels (novice, intermediate, expert) and training settings (LMIC versus HIC), following the development and piloting of a novel educational module for laparoscopic cholecystectomy.

## Materials and methods

### Users

This study took place from May—July 2024 at eight hospitals. The LMIC cohort (*n* = 27) consisted of users from Mbingo Baptist Hospital (Cameroon) (15), Tenwek Hospital (Kenya) (10), Soddo Christian Hospital (Ethiopia) (1), and University of Maiduguri Teaching Hospital (Nigeria) (1). The HIC cohort (*n* = 25) included users from University of California San Francisco (California) (9), University of Michigan (Michigan) (8), Case Western Reserve University (Ohio) (6), University of California San Francisco Fresno (California) (1), and the University of Florida (Florida) (1). Users represented a range of experience levels, including expert laparoscopic surgeons, attending surgeons with minimal laparoscopic experience, general surgery residents at various stages of training, and medical students with minimal or no prior exposure to laparoscopy. For analyses, users were categorized both by their training levels—medical students, resident physicians [House Officer 1 (HO1)—House Officer 6 (HO6)], and attending surgeons—and by their laparoscopic experience. Laparoscopic experience level was classified into three categories: novice (no prior experience as the primary surgeon, intermediate (1–30 cases), and expert (primary surgeon in > 30 laparoscopic cases). All users provided informed consent prior to enrollment. This study received IRB exemption from the University of Michigan’s Institutional Review Board (IRB #HUM00199557).

### Educational module creation

An online case-based learning (CBL) module was developed to facilitate self-directed learning in the management of gallbladder disease, encompassing diagnosis, treatment planning, and post-surgical follow-up. The initial draft of the module was created by a surgical attending from Cameroon to ensure relevance to users and patients in Sub-Saharan Africa. The materials were structured using Bloom’s taxonomy, to promote progressive skill acquisition [17, 18]. Iterative revisions were conducted by a multidisciplinary team, including Cameroonian, Ethiopian, and US general surgeons, as well as a psychometrician and implementation science expert, to ensure clarity and cultural relevance with the module’s educational objectives. The final module was hosted on an open-source web platform optimized for low-bandwidth settings, to enhance accessibility in resource-limited environments.

The module included a demographic survey, a pre- and post-test to evaluate knowledge acquisition, a clinical case scenario on gallbladder disease management, and an expert video demonstrating a step-by-step procedural guide to serve as the operative paradigm. The expert video sequentially demonstrated identification of the anatomy of the gallbladder and surrounding structures, mobilization of the gallbladder with blunt dissection, ligation of the cystic artery and duct with sutures, and removal of the gallbladder from the laparoscopic box trainer.

### Cholecystectomy simulator design & construction

**Fig. 1:**
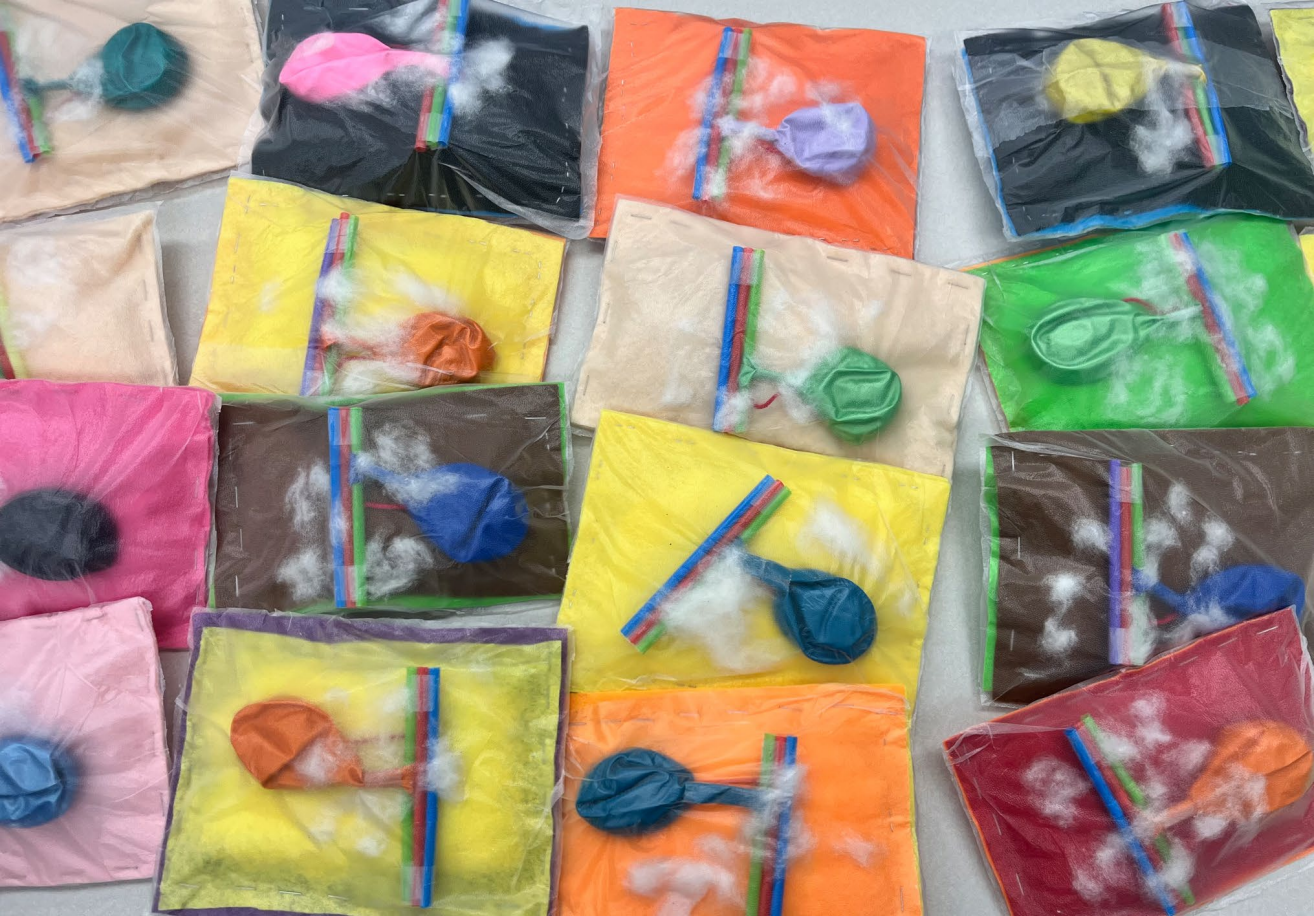
Models for the ALL-SAFE gallbladder module

**Fig. 2:**
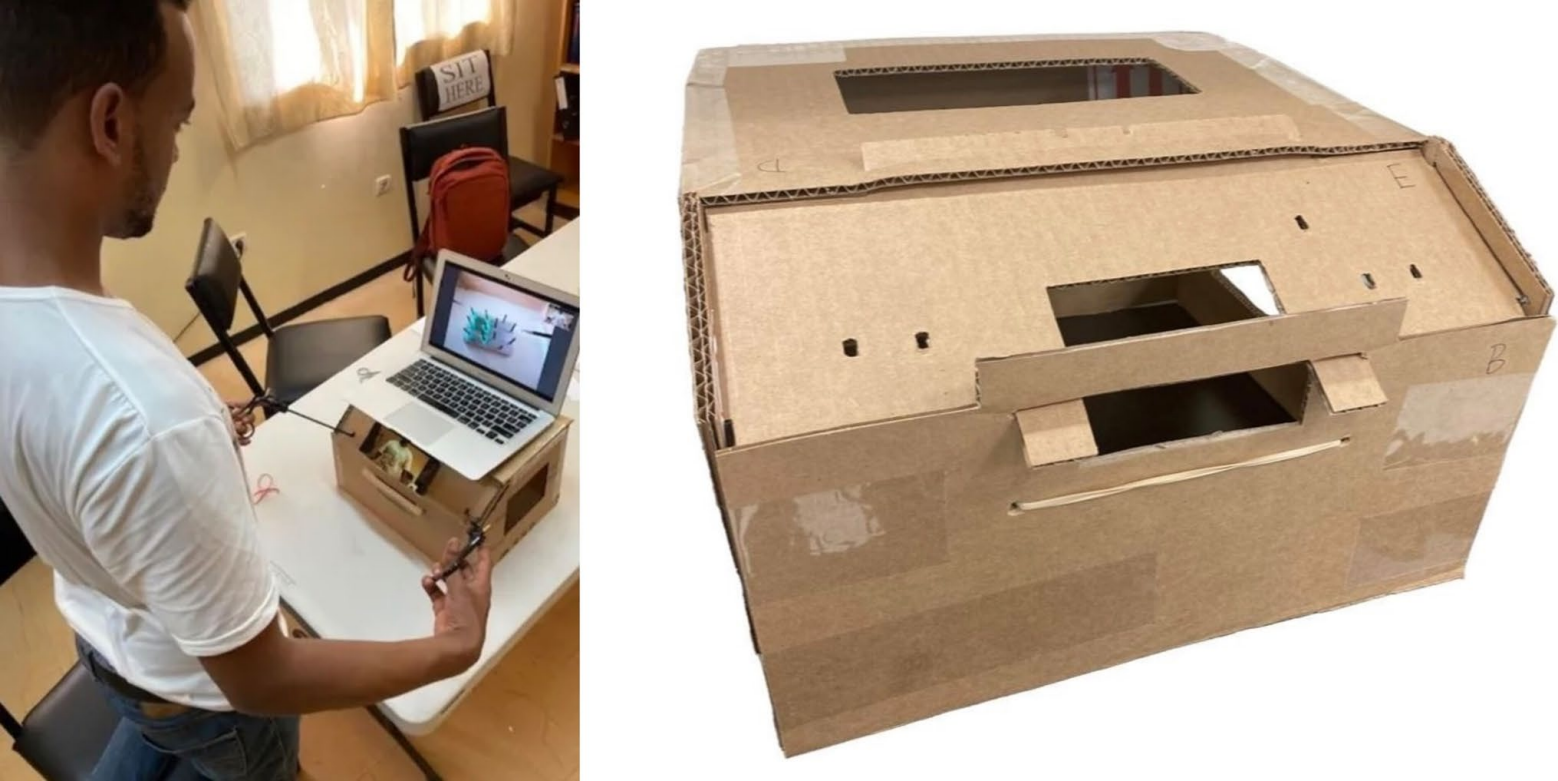
Simulation set-up for the ALL-SAFE platform

The user-assembled cholecystectomy models (Fig. 1) and ALL-SAFE box trainer (Fig. 2**)** were designed to be cost-effective, using readily available materials costing less than $10 USD. The simulation set-up consists of a video-capable cell phone, laptop computer, and Wi-Fi or Bluetooth connectivity for video submission. The mobile phone is used for filming the procedure, and the laptop is used as a monitor to project the recorded video. Necessary laparoscopic instruments for the procedure include: blunt grasper, curved tapered (Maryland) grasper, scissors, needle driver, and multiple 2–0 silk sutures.

### Cholecystectomy verification of proficiency (CHOLE-VOP)

**Fig. 3:**
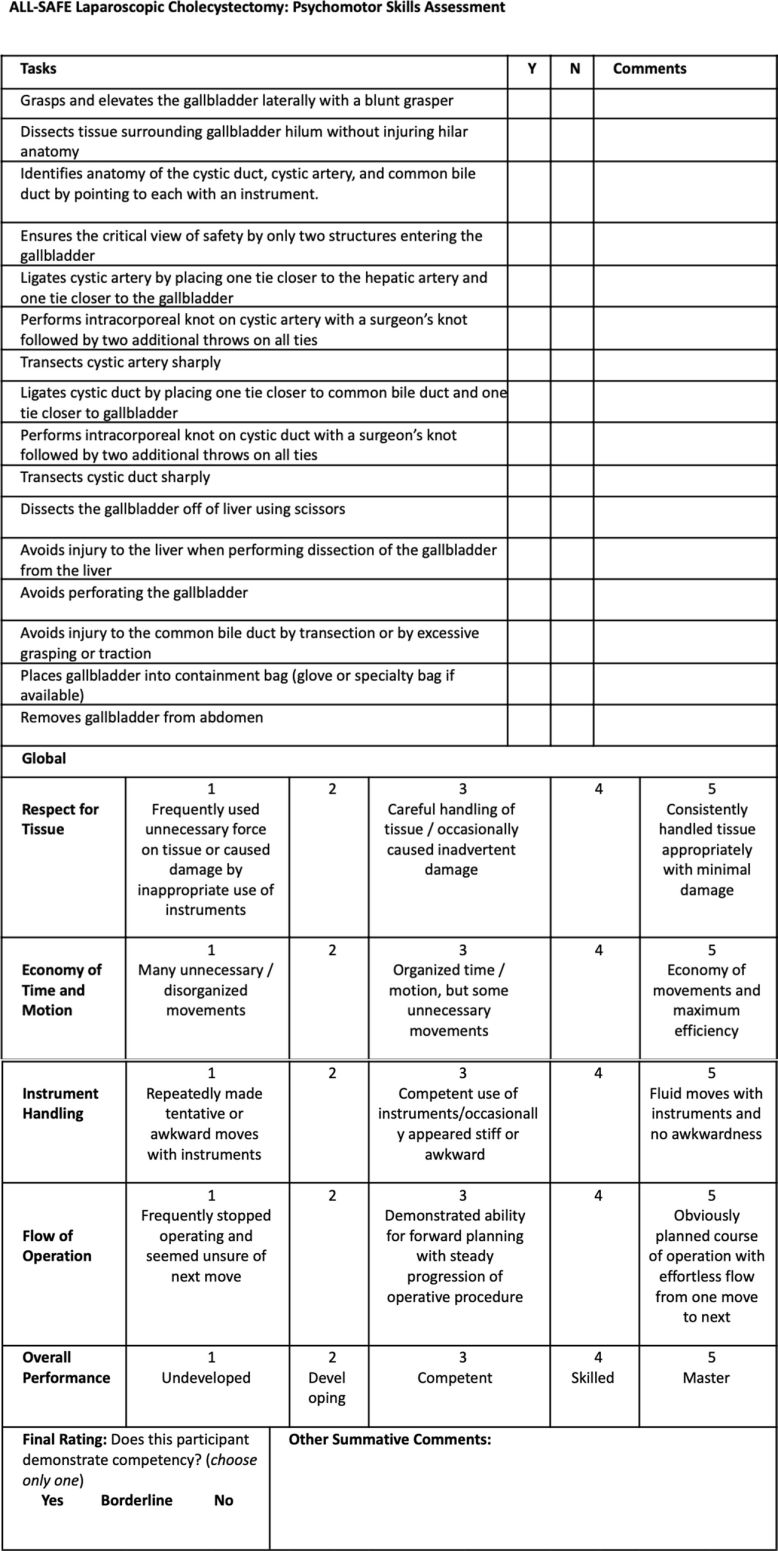
CHOLE-VOP, a verification of proficiency assessment to evaluate cholecystectomy procedural skills

Users were provided with the CHOLE-VOP tool, which functioned as both a procedural guide and a verification of proficiency (VOP) checklist for self-assessment and peer-assessment **(**Fig. 3**)**. Following completion of the module, users recorded a video of their own performance, conducted a self-assessment, and provided peer-assessments for three randomly assigned videos submitted by other users. All assessments were made using the CHOLE-VOP.

The CHOLE-VOP tool was developed through expert consensus, rooted in the Global Rating Scale (GRS) and guided by the American College of Surgeons (ACS) and Association of Program Directors in Surgery (APDS) online curriculum [19]. A Cameroonian co-investigator with prior laparoscopic experience drafted the CHOLE-VOP. This draft underwent multiple rounds of iterative review by the multidisciplinary research team described above, to ensure alignment with the targeted psychomotor skills. A final review by a psychometrician refined the tool further to optimize question clarity and ensure alignment with psychomotor skills critical for competency in laparoscopic cholecystectomy.

The finalized CHOLE-VOP consisted of three components: a 16-item psychomotor skills checklist, a 5-item GRS, and a 3-point final competency rating **(**Fig. 3**)**. The checklist assessed performance in critical steps of laparoscopic cholecystectomy, with items scored as 0 or 2, except for three items addressing complication avoidance, which were scored as 0 or 3 (maximum score = 36). The GRS was scored on a 5-point scale (maximum score = 25). Overall competency was rated on a three-point scale, categorizing users as “Not competent” (1), “Borderline” (2), or “Competent” (3).

### Data analysis

Following confirmation of a non-normal distribution, the CHOLE-VOP’s ability to differentiate between novice, intermediate, and expert performance levels was evaluated using the Kruskal–Wallis test. The same test was used to compare performance between users from LMIC and HIC groups. Given the self- and the peer-assessments utilized the same CHOLE-VOP, the agreement between self- and peer- ratings was studied to evaluate the ability to self-assesss by experience level of rater. Inter-rater agreement was assessed by averaged two-way mixed intraclass correlations (ICC) between novice self-assessments and peer-assessments received from more experienced users (intermediate and expert users). The same comparison was made for intermediate users. Statistical analyses were conducted using SPSS Statistics v.25 (IBM, Armonk, NY) and Facets software v.3.50 (Winsteps.com, Beaverton, OR).

## Results

Fifty-two users completed the ALL-SAFE laparoscopic cholecystectomy module between May and July of 2024 (Table 1). Users included nine medical students, 38 residents (with various levels of training from HO1 to HO6+), one minimally invasive surgery fellow, and four attending surgeons. The LMIC (51.9%) and HIC cohorts (48.1%) were evenly balanced. Prior laparoscopic experience levels were balanced among novice (*n* = 14, no laparoscopic cases), intermediate (*n* = 18, < 30 laparoscopic cases, *M* = 9.2, SD = 6.8), and expert users (*n* = 17, ≥ 30 laparoscopic cases, *M* = 355.9, SD = 544.5). Two users had an unknown experience level and were excluded from analyses comparing users by experience level.

**Table 1:** User demographics for ALL-SAFE laparoscopic cholecystectomy virtual case-based learning module

	Study site		
	Lower- and middle-income country *n* = 27 (51.9%)	High-income country *n* = 25 (48.1%)	Total *N* = 52
*Training level*			
Surgeon	3	1	4
Fellow	0	1	1
Resident	24	14	38
Medical student	0	9	9
*Experience level*			
Expert (> 30 cases)	4	13	17
Intermediate (1–30 cases)	16	2	18
Novice (0 cases)	4	10	14
Unknown	3	0	3

Analysis of the CHOLE-VOP checklist summed scores revealed significant differences in performance scores across experience levels (Table 2). Novice users scored lower (*M* = 28.16 (SD = 7.62) than intermediate users (*M* = 31.33 (3.76), and expert users (*M* = 32.66 (3.43), *P* < 0.001, with high practical effect, *ɳ*^2^ < 0.11. The GRS summed scores (*N* = 13.0 vs *I* = 16.9 vs *E* = 18.6; *P* < 0.001) and the final ratings (*N* = 1.27 vs *I* = 1.64 vs *E* = 1.69; *P* < 0.001) effectively discriminated the experience levels.

**Table 2:** Comparison of psychomotor skill performance ratings across novice, intermediates, and expert users

Performance ratings		Novice (*n* = 14) Mean (SD)	Intermediate (*n* = 18) Mean (SD)	Expert (*n* = 17) Mean (SD)	*P*-value
*Checklist item*					
1	Grasps and elevates the gallbladder laterally with a blunt grasper	1.31 (0.96)	1.29 (0.96)	1.61 (0.80)	0.08
2	Dissects tissue surrounding gallbladder hilum without injuring hilar anatomy	1.75 (0.67)	1.89 (0.46)	1.94 (0.36)	0.12
3	Identifies anatomy of the cystic duct, cystic artery, and common bile duct as part of identifying critical view of safety by pointing to each with an instrument	1.38 (0.93)	1.40 (0.92)	1.71 (0.71)	0.06
4	Ensures the critical view of safety by only two structures entering the gallbladder	1.75 (0.67)	1.78 (0.63)	2.0 (0.0)	0.02
5	Ligates cystic artery by placing one tie closer to the hepatic artery and one tie closer to the gallbladder	1.60 (0.81)	1.92 (0.40)	1.84 (0.55)	0.01
6	Performs intracorporeal knot on cystic artery with a surgeon’s knot followed by two additional throws on all ties	1.49 (0.88)	1.67 (0.75)	1.84 (0.55)	0.04
7	Transects cystic artery sharply	1.82 (0.58)	1.97 (0.23)	1.97 (0.25)	0.04
8	Ligates cystic duct by placing one tie closer to common bile duct and one tie closer to gallbladder	1.49 (0.88)	1.92 (0.40)	1.94 (0.36)	< 0.001
9	Performs intracorporeal knot on cystic duct with a surgeon’s knot followed by two additional throws on all ties	1.53 (0.86)	1.75 (0.66)	1.71 (0.71)	0.21
10	Transects cystic duct sharply	1.71 (0.71)	1.89 (0.46)	1.97 (0.25)	0.02
11	Dissects the gallbladder off of liver using scissors	1.60 (0.81)	1.97 (0.23)	1.74 (0.68)	0.002
12	Avoids injury to the liver when performing dissection of the gallbladder from the liver	2.67 (0.94)	2.75 (0.83)	2.95 (0.38)	0.12
13	Avoids perforating the gallbladder	2.45 (1.17)	2.59 (1.04)	2.71 (0.89)	0.41
14	Avoids injury to the common bile duct by transection or by excessive grasping or traction	2.13 (1.38)	2.84 (0.69)	2.90 (0.53)	< 0.001
15	Places gallbladder into containment bag (glove or specialty bag if available)	1.71 (0.71)	1.81 (0.46)	1.87 (0.50)	0.34
16	Removes gallbladder from abdomen	1.78 (0.63)	1.89 (0.46)	1.97 (0.25)	0.1
	Summed checklist (maximum score = 36)	28.16 (7.62)	31.33 (3.76)	32.66 (3.43)	< 0.001
*GRS*					
1	Respect for tissue	2.64 (1.11)	3.48 (0.91)	3.92 (1.01)	< 0.001
2	Economy of time and motion	2.40 (1.16)	3.27 (1.00)	3.50 (1.11)	< 0.001
3	Instrument handling	2.62 (1.11)	3.27 (1.04)	3.65 (1.07)	< 0.001
4	Flow of operation	2.78 (1.27)	3.49 (0.83)	3.92 (1.01)	< 0.001
5	Overall performance	2.55 (1.14)	3.37 (0.91)	3.63 (0.91)	< 0.001
	Summed GRS (maximum score = 25)	12.98 (5.25)	16.89 (4.40)	18.61 (4.66)	< 0.001
*Overall competency rating*					
	Final rating (maximum score = 3)	1.89 (0.81)	2.53 (0.69)	2.66 (0.54)	< 0.001
	Final rating (maximum score = 2)	1.27 (0.45)	1.64 (0.48)	1.69 (0.46)	< 0.001

Across LMIC and HIC users, summed checklist scores were higher among LMIC users (*M* = 32.41) than HIC users (*M* = 29.35), *P* = 0.008, although little practical effect, *ɳ*^2^ = 0.03 (Table 3**)**. The LMIC users outperformed on intracorporeal suturing and knot tying (Items 5, 6, 8, 9), transection of cystic duct (Item 10), dissection and handling of gallbladder (Items 11, 13), handling the common bile duct (Item 14), and safe removal of the gallbladder (Items 15, 16) (Fig. 3). Similarly, the GRS summed scores (LMIC = 17.5 vs HIC = 15.1; *P* = 0.002) and the final ratings (LMIC = 1.7 vs HIC = 1.5; *P* < 0.001) mirrored these trends, showing higher scores among the LMIC cohort.

**Table 3:** Comparison of psychomotor skill performance ratings across high-income countries (HIC) and lower- and middle-income countries (LMIC)

Performance ratings		LMIC (*n* = 27) Mean (SD)	HIC (*n* = 25) Mean (SD)	*P*-value
*Checklist item*				
1	Grasps and elevates the gallbladder laterally with a blunt grasper	1.48 (0.88)	1.32 (0.95)	0.22
2	Dissects tissue surrounding gallbladder hilum without injuring hilar anatomy	1.89 (0.45)	1.84 (0.55)	0.43
3	Identifies anatomy of the cystic duct, cystic artery, and common bile duct as part of identifying critical view of safety by pointing to each with an instrument	1.46 (0.89)	1.53 (0.86)	0.62
4	Ensures the critical view of safety by only two structures entering the gallbladder	1.81 (0.59)	1.88 (0.48)	0.37
5	Ligates cystic artery by placing one tie closer to the hepatic artery and one tie closer to the gallbladder	1.94 (0.36)	1.67 (0.75)	0.002
6	Performs intracorporeal knot on cystic artery with a surgeon’s knot followed by two additional throws on all ties	1.78 (0.62)	1.57 (0.83)	0.04
7	Transects cystic artery sharply	1.96 (0.29)	1.90 (0.45)	0.27
8	Ligates cystic duct by placing one tie closer to common bile duct and one tie closer to gallbladder	1.94 (0.36)	1.67 (0.75)	0.011
9	Performs intracorporeal knot on cystic duct with a surgeon’s knot followed by two additional throws on all ties	1.78 (0.62)	1.57 (0.83)	0.001
10	Transects cystic duct sharply	1.96 (0.23)	1.90 (0.45)	0.012
11	Dissects the gallbladder off of liver using scissors	1.96 (0.29)	1.63 (0.78)	< 0.001
12	Avoids injury to the liver when performing dissection of the gallbladder from the liver	2.84 (0.68)	2.75 (0.83)	0.43
13	Avoids perforating the gallbladder	2.77 (0.80)	2.41 (1.19)	0.016
14	Avoids injury to the common bile duct by transection or by excessive grasping or traction	2.90 (0.53)	2.41 (1.20)	< 0.001
15	Places gallbladder into containment bag (glove or specialty bag if available)	1.94 (0.36)	1.67 (0.75)	0.002
16	Removes gallbladder from abdomen	1.96 (0.29)	1.81 (0.58)	0.04
	Summed checklist (maximum score = 36)	32.41 (2.86)	29.35 (6.69)	0.008
*GRS*				
1	Respect for tissue	3.56 (0.94)	3.21 (0.91)	< 0.001
2	Economy of time and motion	3.40 (1.01)	2.80 (1.25)	0.032
3	Instrument handling	3.43 (1.00)	2.99 (1.23)	0.055
4	Flow of operation	3.67 (0.89)	2.95 (1.18)	< 0.001
5	Overall performance	2.55 (1.14)	3.37 ( 0.91)	0.040
	Summed GRS (maximum score = 25)	17.54 (4.31)	15.14 (5.7)	0.002
*Overall competency rating*				
	Final rating (maximum score = 3)	2.58 (0.61)	2.21 (0.48)	< 0.001
	Final rating (maximum score = 2)	1.65 (0.48)	1.46 (0.59)	0.008

Inter-rater agreement suggested mixed rater agreement across CHOLE-VOP domains. Across all components of CHOLE-VOP, novice users had significantly higher self-assessment scores than peer-assessments (Table 4). Spearman rank correlations between "self" and "peer" assessments for users of all experience levels showed moderate agreement for summed checklist scores (*ρ* = 0.52), poor agreement for summed GRS (*ρ* = 0.44), and moderate for final rating (*ρ* = 0.60). When comparing scores between novice self-assessment scores with peer-assessment scores given by intermediate or expert users, agreement was poor for the checklist (0.24), moderate for the GRS (0.55), and moderate for the final rating (0.65). Intermediate users demonstrated good agreement on the checklist (0.79) and moderate agreement on the GRS (0.52) and the final rating (0.45).

**Table 4:** Inter-rater agreement between novice self-rating and peer-ratings from others and intermediate self-rating and peer-ratings

Domain	Novice self-rating Mean (SD)	Intermediate/expert rating of novice Mean (SD)	ICC	95% CI	Intermediate self-rating Mean (SD)	Expert rating of intermediate Mean (SD)	ICC	95% CI
Checklist Summed	30.67 (5.38)	28.40 (8.82)	0.24	− 0.68 to 0.65	32.87 (2.86)	31.89 (3.85)	0.67	0.35 to 0.83
Respect for Tissue	2.92 (1.16)	2.80 (1.22)	0.68	0.28 to 0.85	3.74 (0.73)	3.52 (1.07)	0.42	− 0.15 to 0.71
Economy of Time/Motion	2.75 (1.29)	2.48 (1.23)	0.31	− 0.52 to 0.68	3.19 (0.87)	3.40 (1.11)	0.60	0.20 to 0.79
Instrument Handling	2.92 (1.16)	2.68 (1.25)	0.48	− 0.14 to 0.76	3.42 (0.76)	3.50 (1.10)	0.35	− 0.29 to 0.67
Flow of Operation	3.25 (1.29)	2.72 (1.34)	0.53	− 0.06 to 0.79	3.55 (0.93)	3.68 (0.94)	0.51	0.04 to 0.76
Overall Performance	2.75 (1.14)	2.64 (1.25)	0.48	− 0.15 to 0.76	3.42 (0.67)	3.50 (0.95)	0.50	0.00 to 0.75
Global Summed	14.58 (5.47)	13.32 (5.90)	0.55	0.01 to 0.79	17.32 (3.40)	17.60 (4.91)	0.54	0.09 to 0.77
Final rating	2.00 (0.85)	2.04 (0.79)	0.65	0.23 to 0.84	2.68 (0.54)	2.60 (0.64)	0.53	0.07 to 0.76

## Discussion

This study demonstrates the successful pilot of a simulation-based training module for laparoscopic cholecystectomy among users of varied training level and laparoscopic experience, in HIC and LMIC settings. The CHOLE-VOP tool effectively discriminated laparoscopic experience level. The LMIC cohort outperformed the HIC cohort on the summed checklist and skill parameters related to intracorporeal suturing and tissue handling. Inter-rater agreement between self- and peer-assessment was limited, especially for the the checklist, but improved with increasing experience level of the user conducting the self-assessment.

This platform highlights the value of peer-assessments as both an assessment and learning tool in building a foundation for psychomotor skills [21–23]. Self-assessments, particularly among novices, have been criticized for potentially fostering a false sense of competency, making them unreliable as standalone assessment tools [24]. Peer-assessments, from multiple reviewers of varied experience levels, are more likely to improve assessment quality and objectivity, compared to a single self-assessment or expert review. The act of performing peer-assessments may also offer even greater educational benefits, as applying the CHOLE-VOP tool to evaluate others requires a deeper understanding of its criteria and an ability to discern the failure to meet criteria. Under the train-the-trainer model that underpins self-learning, the ability to analyze and interpret others’ performances is an essential part of learning, as it enhances both teaching skills and learner confidence.

The LMIC cohort demonstrated superior performance in intracorporeal knot tying, transection of the cystic duct, and tissue handling. Intracorporeal suturing and knot tying are fundamental laparoscopic techniques, but their routine use has declined in HICs due to the widespread adoption of energy-sealing devices, surgical clips, staplers, and barbed sutures [20]. This shift in standard practice may explain lower scores among HIC users on select CHOLE-VOP items. In contrast, LMIC surgeons, who may have limited access to disposable surgical devices, may develop stronger proficiency in these specific techniques. These findings highlight the impact of evolving practices on surgical education and underscore the need for training that ensures competency in diverse resource settings.

Novices tended to overestimate their performance, while intermediate users, with likely more experience evaluating others’ performance as well, showed higher agreement between self- and peer-assessments received from expert users. This pattern aligns with the Dunning-Kruger effect, wherein less experienced users lack the metacognitive ability to accurately evaluate their own performance or point out deficiencies [25, 26]. The dual burden of limited insight into deficits coupled with lower level of proficiency, is magnified in self-learning, especially when the expert is absent [29, 30]. While self-assessment accuracy tends to improve with experience, structured peer and expert evaluations remain crucial at all levels to calibrate skill perception [27].

While the platform provides an opportunity for practicing laparoscopy in a safe environment, completing modules or achieving higher scores on the CHOLE-VOP does not equate to readiness for live cases. While self-assessments are known to increase self-efficacy, overconfidence, particularly among novice users, may lead to inappropropriate or premature utilization of skills in the OR, posing risk to patient safety [28]. Continued practice without supervision checkpoints can also lead to the development of anti-skills, which undermine safe practices. Thus, simulation-based learning must build on a strong existing foundation of cognitive and psychomotor proficiency and must be followed by the appropriate in-person observership and guided intraoperative practice. Confidence and interest stimulated by engagement on the platform should be re-directed to pursuit of opportunities for skill growth beyond the platform, whether it be direct observation or assistance in the OR.

### Limitations

This study has several limitations that should be considered. First, the small number of users and uneven distribution of training level and laparoscopic experience levels across cohorts, may affect the generalizability of the findings. In addition, all users included were invited from a nascent global network of collaborators within PAACS, which may limit its relevance and applicability to other training programs and learning environments. Consequently, all users may have had pre-existing familiarity with the simulation-based platform and may have participated in other prior educational modules within the same simulation-based training platform.

Future studies should aim to recruit users who are naïve to the platform and represent varying ranges of skill levels, from different institutions. In order to have more balanced cohorts, it would be beneficial to recruit users from other HIC settings outside of the US. Further, future work can build on the established construct validity to evaluate whether laparoscopic cholecystectomy skills gained from the ALL-SAFE platform are associated with improved live laparoscopic cholecystectomy performance.

## Conclusion

This pilot study provides evidence supporting the CHOLE-VOP assessment tool as a method for teaching and evaluating laparoscopic cholecystectomy through a low-cost, simulation-based platform. Piloted across both LMIC and HIC training environments and among users of varying experience levels, CHOLE-VOP successfully distinguished user performance based on experience level and revealed trends of higher performance among LMIC users. The low concordance between self- and peer-assessments on the checklist, particularly among novice users, highlights the need for a multicomponent assessment approach.
